# Pharmacists’ Willingness to Offer Vaccination Services: A Systematic Review and Meta-Analysis

**DOI:** 10.3390/pharmacy12040098

**Published:** 2024-06-26

**Authors:** Arit Udoh, Desak Ernawati, Ifunanya Ikhile, Asma Yahyouche

**Affiliations:** 1 Faculty of Health & Life Sciences, University of Exeter, Exeter EX1 2LU, UK; 2 Department of Pharmacology and Therapy, Universitas Udayana, Denpasar 80234, Bali, Indonesia; dernawati001@gmail.com; 3 School of Medicine, University of Nottingham, Nottingham NG7 2UH, UK; ifunanya.ikhile1@nottingham.ac.uk; 4 School of Pharmacy, College of Medical and Dental Sciences, University of Birmingham, Edgbaston, Birmingham B15 2TT, UK; a.yahyouche@bham.ac.uk

**Keywords:** vaccination, immunization, vaccine, pharmacist-based vaccination (PBV), vaccination willingness

## Abstract

Pharmacy-based vaccination (PBV) services increase coverage and enhance access to lifesaving vaccines. This systematic review assessed the proportion of pharmacists willing to offer PBV services. PubMed/MEDLINE, CINAHL, EMBASE and Scopus electronic databases were searched from inception to identify relevant literature. Google scholar and other sources of grey literature was also searched. The literature findings were synthesized narratively, and via a random-effects meta-analysis. Risk of bias was evaluated using nine quality assessment criteria adapted from the Joanna Briggs Institute checklist for prevalence studies. The review protocol is registered on PROSPERO (REF: CRD42021293692). In total, 967 articles were identified from the literature search. Of this, 34 articles from 19 countries across 5 WHO regions were included in the review. No article from the Western Pacific WHO region was identified. Most of the included studies (n = 21, 61.8%) showed an overall low risk of bias. None showed a high risk of bias. Pooled willingness for PBV services was 69.45% (95% CI: 61.58–76.33; n total pharmacists = 8877), indicating that most pharmacists were willing to offer the service, although nearly a third were not. Pharmacists’ willingness was highest in the Americas (71.49%, 95% CI: 53.32–84.63, n pharmacists = 3842) and lowest in the African region (58.71%, 95% CI: 45.86–70.46, n pharmacists = 1080) although the between-group difference was not statistically significant across the WHO regions (Q = 3.01, df = 4, *p* < 0.5567). Meta-regression showed no evidence (R^2^ = 0%, *p* = 0.9871) of the moderating effect of the type of vaccine assessed, PBV service availability, sampling technique and the study risk of bias. These findings show that most pharmacists are willing to offer PBV services; however, strategies that will enhance greater involvement in service provision are needed.

## 1. Introduction

Globally, pharmacists’ scope of practice has expanded to include authorization for vaccination services [1]. Pharmacy-based vaccination (PBV) services are available in 56 countries and territories, representing an increase from the 34 countries reported in a previous survey conducted in 2020 [1]. Influenza, COVID-19, Tdap (tetanus, diphtheria and pertussis) and hepatitis B vaccines are the most prevalent vaccinations offered within PBV services [1,2,3]. Prior to the onset of the COVID-19 pandemic, pharmacists were authorized to offer specific vaccines to defined population groups such as the elderly and those at risk [4,5,6]. In addition, disparities existed across countries on whether the vaccines offered within PBV services were provided with or without a prescription [7]. With the onset of the COVID-19 pandemic and the subsequent mass vaccination programs to curb the disease, authorization for PBV services further expanded globally [8,9,10,11,12]. Pharmacists are now authorized to provide PBV services to the general population in several countries across Europe, the Western Pacific and in North and Latin America [1,8,9,10,11,12].

The contributions of PBV services to national and global vaccination programs are well documented [2,13,14,15,16,17]. Studies show that pharmacists’ role as vaccinators, vaccine advocates and educators as well as their vaccine supply responsibilities increase vaccination coverage and population uptake [13,18]. Increase in vaccine accessibility, greater convenience, lower risk of infection and expanded access for vulnerable and at-risk populations are other benefits of PBV services reported in the literature [14,17,19]. Research from the United States demonstrates economic benefits, which include the lower cost of PBV services compared to vaccination services provided in traditional medical settings such as in general practices and primary care facilities [20,21,22]. Service evaluation studies also indicate high levels of patient satisfaction, further emphasizing the need for continued PBV service availability [19,23,24,25,26]. However, research shows disparity in pharmacists’ willingness to offer PBV services. In this paper, pharmacists’ willingness is defined as the proportion of pharmacists who are willing to offer PBV services. In countries such as the United States and Canada where PBV services are well established, studies show pharmacists’ willingness ranging from 52 to 98% [27,28,29,30]. In other countries where the service is under consideration or newly implemented, research shows pharmacists’ willingness ranging from 45 to 83% [31,32,33]. Factors such as poorly defined regulations, training unavailability, high workload and poor reimbursement are some of the barriers influencing pharmacists’ willingness to offer PBV services [34]. For instance, reports indicate that several of the vaccines offered within PBV services are not reimbursable or covered by available health insurance plans [30,34,35,36,37,38,39,40,41]. Inadequate staffing resulting in a high workload and limited PBV training availability or certification programs are other reported barriers to pharmacists’ involvement in PBV service provision [34]. In addition, willingness differed relative to pharmacists’ area of practice, experience, practice location alongside other factors that include type of vaccine offered, specific population group covered and cost [30,34,35,36,37,38,39,40,41]. For example, more pharmacists in urban areas were willing to offer and obtain the required certification for PBV services compared to those in rural practices [35]. Other reports also show that pharmacists with fewer than ten years of experience were willing to offer the service compared to those with longer experience [29,33,40]. As more countries aim to implement and/or expand PBV services, published evidence on the extent of pharmacists’ willingness to offer the service is required. This global review aims to explore pharmacists’ willingness to offer vaccination services.

## 2. Materials and Methods

### 2.1. Search Strategy

A systematic review of the published literature was conducted. PubMed/MEDLINE, CINAHL, EMBASE and Scopus electronic databases were searched for the relevant literature. Each database was searched from inception to November 2023. Free text search of Google Scholar and four electronic sources of the grey literature (Scirus, Mednar, CiteSeerX and OpenGrey) was also conducted. Pharmacy-related journals (details provided in Appendix A) and bibliographies of the identified literature were also searched. Key words used for the database searches were as follows: ‘Pharmacy’, ‘Pharmacist’, ‘Pharmacist-led vaccination’, ‘pharmacy-based vaccination’, ‘perceptions’, ‘vaccine’, ‘vaccine acceptance’, ‘vaccination willingness’, ‘immunization’ and ‘inoculation’. The key words were combined using the Boolean operators “OR” and “AND” with use of truncation where appropriate to ensure inclusion of relevant Medical Subject Headings (MeSH) terms. There was no restriction imposed on language, vaccine type, year of publication and country. The Medline database search strategy that was adapted for the other databases is presented in Appendix A. The protocol for this review is registered on PROSPERO with reference number CRD42021293692.

### 2.2. Study Selection

**Figure 1:**
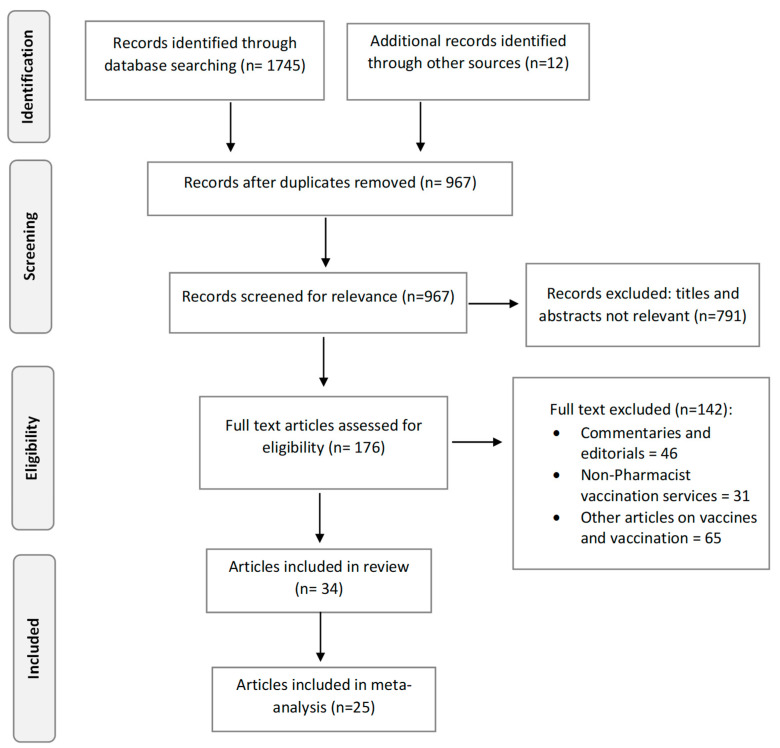
Schematic of literature selection process using the Preferred Reporting Items for Systematic Reviews and Meta-Analysis (PRISMA).

Two authors (AU and DE) independently screened the titles and abstract identified from the literature search. Screening included assessing the identified literature to determine its relevance to the review objectives. Full-text screening of articles that appeared relevant was then conducted with respect to the inclusion criteria. Thereafter, two authors (AU and DE) independently reviewed and validated the literature selection with the outcome of the screening process compared for consistency by the other two remaining authors (II and AY). Any observed discrepancy was resolved through further discussion between the authors and subsequent consensus. Data extraction occurred under the following headings: author(s), year of publication, country, World Health Organization (WHO) region, sampling strategy, study design, vaccine type evaluated, proportion of pharmacists willing to offer the service and total sample size. Primary research articles specifically addressing pharmacists’ willingness to offer vaccination services were included. Excluded studies were those involving vaccination services by other healthcare providers. Also excluded were editorials, commentaries and other publications that did not meet the pre-defined inclusion criteria. The data extraction form designed for this review was piloted in 10% of the studies to be extracted. At the end of the pilot phase, a change to the extraction form was not required and the remaining studies were subsequently extracted. A schematic of the literature selection process is presented in Figure 1 using the Preferred Reporting Items for Systematic Reviews and Meta-Analyses (PRISMA) [42].

### 2.3. Analysis

The findings from the included studies were summarized narratively in line with the review objectives. A meta-analysis was also conducted using the R meta prop package in RStudio(v4.3.1) [43]. The random-effects (RE) meta-analytical model computed using the inverse variance method assessed the proportion of pharmacists per study that reported willingness to provide the service. The outcome of this analysis is presented using a Forest plot. Between-study heterogeneity was evaluated using the Higgins I-squared (I^2^) statistic with values of 25%, 50% and 75% and above indicating low, moderate and high heterogeneity, respectively [44]. Sensitivity analyses were conducted to assess the robustness of the pooled estimates via meta-regression, subgroup analysis and exclusion of outliers. Outliers were defined as studies with 95% CI outside the range of the overall pooled estimate. The subgroups defined a priori included period of publication (pre-COVID vs. during vs. post), type of vaccine assessed (COVID-19 vs. flu vs. non-specific), PBV services available during study (yes vs. no), WHO region (Eastern Mediterranean (EMR) vs. Southeast Asia (SEAR) vs. Africa (AFR) vs. the Americas (AMR) vs. Europe (EUR) vs. Western Pacific (WPR), sampling technique (random vs. non-random) and the overall risk of bias classification (low vs. moderate vs. high). Publication bias was evaluated using a contour-enhanced funnel plot and confirmed quantitatively via the Eggers test [45,46]. The Duval and Tweedie trim-and-fill procedure alongside a *p*-curve were used to correct for publication bias in the presence of funnel plot asymmetry [47,48]. For ease of interpretation, the proportions in this review are reported as percentages.

### 2.4. Quality Assessment and Risk of Bias

Risk of bias was evaluated using nine quality assessment criteria adapted from the Joanna Briggs Institute checklist for prevalence studies [49]. The nine quality assessment criteria are presented in Table 1. Each criterion received a score of zero if there was a quality concern (high risk) or 1 if none (low risk). Where a criterion was not reported or unclear, a score of zero was entered. A score of zero was also entered when the response rate was less than 50%, and or where this was not reported in the study. Total quality score of 0–3 indicated a high risk of bias, 4–6 showed a moderate risk, while scores of 7 and above suggested a low risk. The maximum total quality score possible was 9. The outcome of the quality assessment is presented using the risk-of-bias visualization package in R [50].

## 3. Results

### 3.1. Characteristics of the Selected Literature

In total, 1745 articles were identified from the literature search. On deduplication, this included 967 unique titles that also included 12 articles from the manual search of the bibliographies of the identified literature. After screening the titles and abstract for relevance, 791 articles were excluded. The full text of 176 papers was then further screened against the review inclusion and exclusion criteria. At the end of the literature search and selection process, 34 articles were identified and selected for inclusion. This included one article that reported research conducted in four countries [31]. The 34 studies selected for this review were conducted in 19 countries across 5 WHO regions (Table 2). The selected literature included eight studies from the United States [3,27,28,30,34,40,51,52]; five from Canada [29,35,36,37,53]; three from Jordan [41,54,55]; three from Nigeria [56,57,58]; two each from Lebanon [38,59], Malaysia [60,61] and Indonesia [62,63]; and one each from Estonia [64], France [65], Italy [66], Austria [33], Serbia [31], Bulgaria [31], Albania [31], Saudi Arabia [32], Romania [31], Poland [39], Ethiopia [67] and Thailand [68]. The study by Turcu-Stiolica et al. reported research conducted in four countries: Romania, Serbia, Albania and Bulgaria [31]. No study from the Western Pacific region was identified for inclusion. A total of 25 of the 34 included articles quantified pharmacists’ willingness to offer PBV services and these were included in a meta-analysis (Figure 1).

The majority (n = 32, 94.1%) of the included studies were surveys. The remaining two studies were semi-structured interviews where participants were explicitly asked whether they were willing to offer PBV services [52,54] (Table 2). The sample size in the survey studies ranged from 68 to 1777 pharmacists, while the two interview studies ranged from 23 to 40 pharmacists (Table 3). Although the majority (n = 32, 94.1%) of the studies assessed community pharmacists’ willingness to offer vaccination services, two assessed a mix of pharmacists from multiple practice areas or roles [27,58]. Most of the studies explicitly assessed whether pharmacists were willing to offer PBV services, while one study by Carpenter et al. assessed pharmacists’ readiness to provide the service [30]. The proportion of pharmacists who expressed readiness to provide PBV services was extracted in the Carpenter et al. study given that this implied willingness [30]. A separate study by Kummer et al. assessed the number of pharmacists who had undertaken certification to provide the service, and these data were also extracted as they implied willingness [51]. More than half (n = 19, 55.9%) of the included studies were conducted during the COVID-19 pandemic between 2020 and 2023, with the remaining conducted prior (Table 2 and Table 3). No post-pandemic study (that is a study conducted after May 2023) was identified for inclusion. One study assessed pharmacists’ willingness to offer a Human Papilloma virus (HPV) vaccination service [66] while COVID-19 [30,31,41,54] and influenza vaccine services [35,36,38,53,64,65] were each assessed in six separate studies, respectively. Most (n = 21, 61.8%) of the included studies did not specify the type of vaccine or vaccination service assessed (Table 2 and Table 3). The majority (n = 19, 55.9%) of the included studies assessed pharmacists’ willingness after the implementation of PBV services in the respective countries [3,27,28,29,30,34,35,36,37,40,41,51,52,53,54,55,64,65,68], while the remaining assessed this prior to service availability (Table 2 and Table 3).

**Figure 2:**
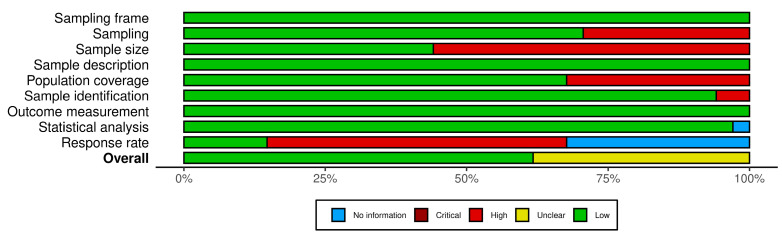
Risk-of-bias summary plot.

**Figure A1:**
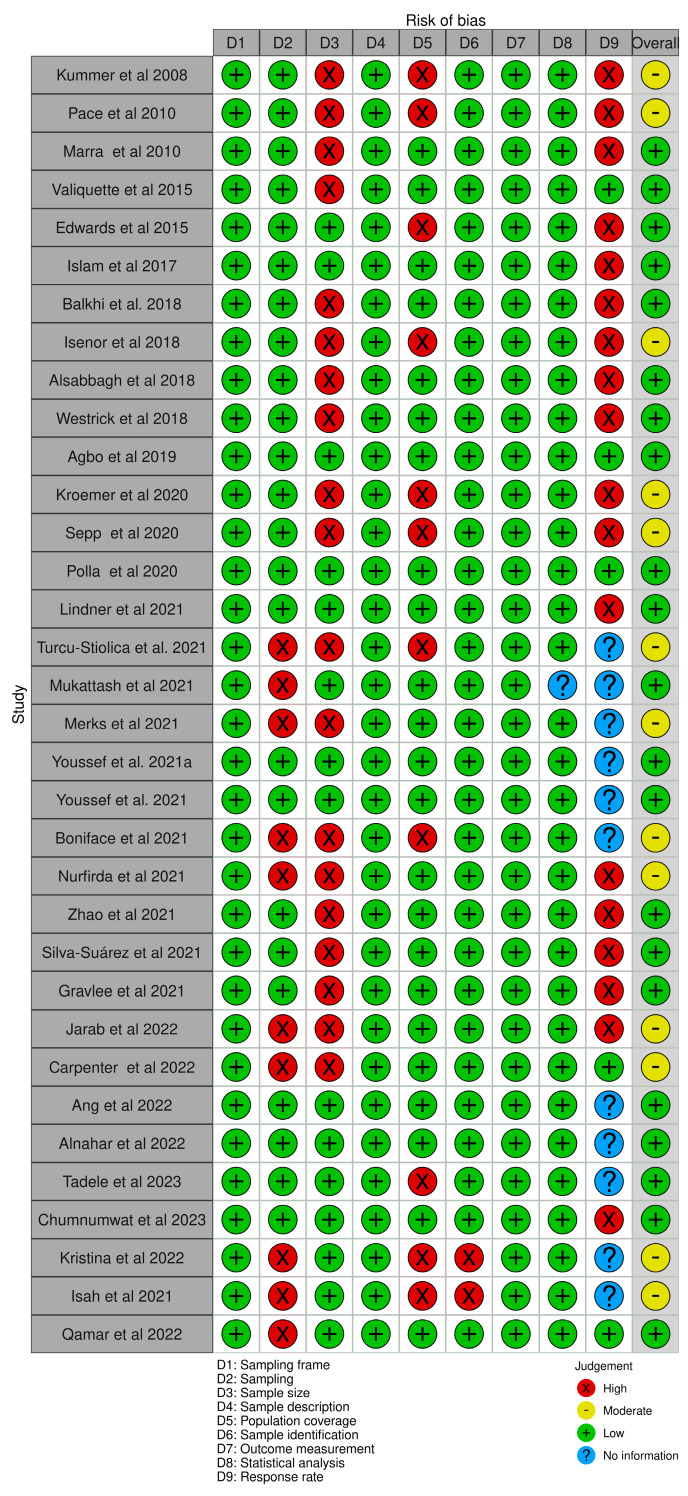
Risk-of-bias traffic light plot of the 34 included studies [3,27,28,29,30,31,32,33,34,35,36,37,38,39,40,41,51,52,53,54,55,56,57,58,59,60,61,62,63,64,65,66,67,68].

Quality assessment conducted across nine domains showed most (n = 21, 61.8%) of the included studies with an overall low risk of bias (quality score = 7–8) [3,27,28,32,33,35,36,37,38,40,52,53,54,55,56,59,60,61,66,67,68]. About a third (n = 13, 38.2%) of the studies had a moderate risk of bias (quality score = 6) [29,30,31,34,39,41,51,57,58,62,63,64,65], while none showed a high risk of bias. Sources of bias related to the sampling process, sample size and low response rate. Most (n = 27, 79.4%) of the studies utilized a non-probabilistic sample with limited information on the justification for the reported sample size. The response rate was not reported in more than half (n = 19, 55.9%) of the included studies. Where the response rate was reported, this was generally low, at less than 50% in about a third (n = 13, 38.2%) of the studies (Table 3). The risk-of-bias summary plot for the included studies is presented in Figure 2 while the outcome of the quality assessment per study is presented using a traffic light plot (see Figure A1 in Appendix A).

### 3.2. Overview of Pharmacists’ Willingness

The proportion of pharmacists who were willing to offer PBV services as reported in the 34 included studies ranged from 22 to 98% (Table 3). Of this number, only four studies reported willingness that was less than 50% overall and these were conducted in Bulgaria [31], the United States [51], Nigeria [58] and Thailand [68]. Disparities in willingness were observed within and across countries, as well as across the respective WHO regions (Table 3). Willingness across the WHO regions ranged from 45 to 90.8% across the nine European countries represented [31,33,39,64,65,66]; 64.5–82.6% in countries represented in the EMR [38,41,54,55,59]; 47.70–74% in the countries in Africa [56,57,58,67] and 42.2–89.2% in the Southeast Asia countries [60,61,62,63,68]. Willingness was at least 75% or more in 9 of the 13 studies included from the Americas [3,28,29,30,34,35,36,37,52]. Of the four remaining studies from the AMR [30,37,53], only one reported willingness of less than 50% and this was the study by Kummer et al. [51]. Although most of the AMR studies were conducted prior to the COVID-19 pandemic, three were carried out between 2020 and 2022 and reported willingness of 52–79% [27,28,30]. This suggested that the reported disparity in willingness observed across the AMR was unlikely to be related to the year the study was conducted or published [27,28,30]. Within-country disparity in willingness was also observed in relation to practice location with more pharmacists in urban areas willing to offer the service compared to those in rural practices [35,59,63]. One study showed that pharmacists in an urban location were twice as likely to be willing to offer [63] and to be certified for PBV services [35]. This contrasted with a study from Malaysia, which showed that more of the pharmacists in rural areas were willing to offer the service compared to the urban practitioners [60].

More pharmacists with fewer than ten years of experience were willing to offer the service compared to those with longer experience [29,33,40,59,62,64]. This finding was reported in studies conducted in Lebanon [59], Estonia [64], the United States [40], Canada [29], Austria [33] and Indonesia [62], suggesting that it was unlikely to be solely related to country context. While pharmacists generally reported possessing limited knowledge about vaccines [27,29,39,62,66] including for mandatory vaccinations [66], the more experienced were even less likely to possess the appropriate knowledge [59,66]. This contrasted with the study by Ang et al., which reported adequate knowledge to provide the service for most of the pharmacists surveyed in Malysia [60]. Adequate knowledge about the vaccines and vaccination correlated with willingness to offer PBV services [61,67]. More independent pharmacists were willing to offer the service compared to chain pharmacists [31,41,52]. Other studies, however, showed independent pharmacists as less likely to obtain the certification needed to provide the service compared to those in chain pharmacies [35,39,40]. Willingness varied in relation to specialization with more specialist pharmacists willing than non-specialist to offer the service [31]. More pharmacists were willing to offer travel, flu and pandemic vaccines compared to routine vaccinations [37]. Most were also interested in vaccinating adults or adolescents rather than children [33,36].

Pharmacists preferred to offer PBV services by appointment only [36], or in combination with walk-ins [35,52] and/or extended opening hours in the evenings and at the weekends [36]. In addition, pharmacists were willing to offer the service in other locations including at mass vaccination clinics, primary care sites and care homes [36]. Although PBV service availability was shown to increase coverage, the service was often provided below capacity with the potential to vaccinate more [52].

The lack of vaccination training was a key factor reported as limiting pharmacists’ involvement and willingness to offer PBV services [31,32,51,54,56,57,60]. However, while undertaking vaccination training and being certified correlated with willingness to offer the service [34,36,68], one study showed that many pharmacists failed to maintain their certification, suggesting that training alone does not guarantee continued involvement in service provision [34]. Patient safety concerns were also reported [32,58,60], though a study by Isenor et al. showed very low risk of adverse events with PBV services [29]. Of note were the findings from two studies that showed that fewer than a third of surveyed pharmacists were familiar with procedures for reporting or managing an adverse event, such as anaphylaxis, with the majority indicating they lacked this knowledge [36,68].

### 3.3. Pooled Estimate of Pharmacists’ Willingness

**Figure 3:**
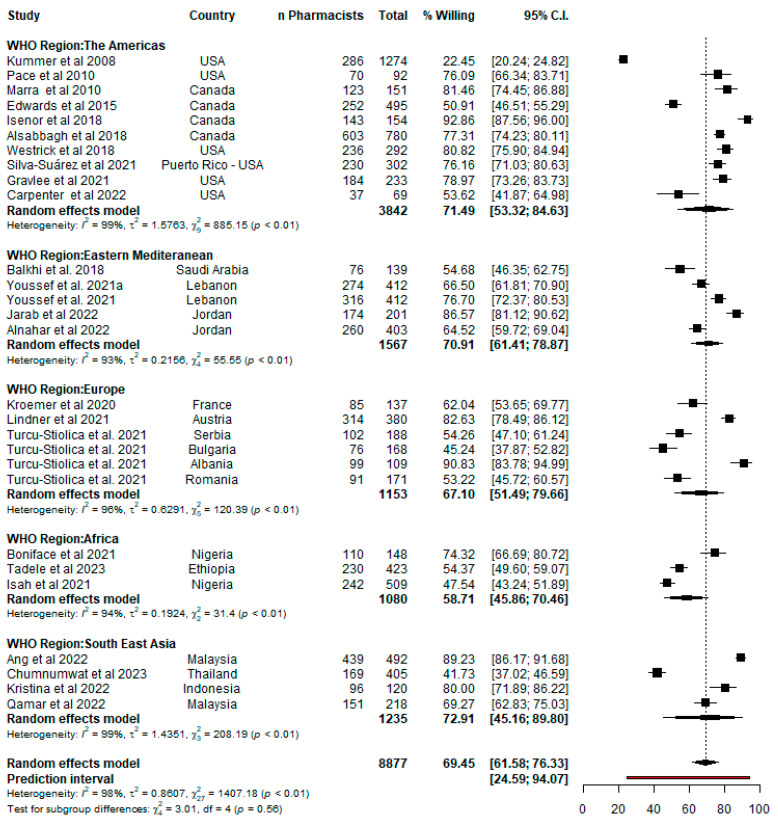
Forest plot of pharmacists’ willingness per study and WHO region.

Twenty-five surveys included in this review quantified pharmacists’ willingness to offer PBV services. This included the study by Turcu et al. that reported willingness across four European countries: Bulgaria, Romania, Serbia and Albania [31]. The literature by Turcu et al. was extracted and included per country in the meta-analysis to yield a total of 28 studies. The meta-analysis included a total of 8877 pharmacists and showed a pooled willingness of 69.45% (95% CI: 61.58–76.33) with high between-study heterogeneity (I^2^ = 98.1%, *p* < 0.0001) observed (Figure 3). Further analysis showed there was no statistically significant between-group difference across the WHO regions (Q = 3.01, df = 4, *p* = 0.5567) (Table 4), although the pooled willingness was generally highest in the Americas (71.49%, 95% CI: 53.32–84.63, I^2^ = 99%) and lowest in the African region (58.71%, 95% CI: 45.86–70.46, I^2^ = 93.6%). When the two studies that implied willingness were removed from the meta-analysis [30,51], the overall pooled estimate increased slightly to 71.48% (95% CI: 65.52–76.78, n pharmacists = 7534) with heterogeneity remaining high at 96.4% (*p* < 0.0001). The between-group difference across the WHO regions was also non-significant (Q = 6.77, df = 4, *p* = 0.1486).

### 3.4. Sensitivity Analysis of Pooled Estimate

Sensitivity analysis conducted to assess the robustness of the pooled estimate identified 13 studies [29,31,32,33,41,51,53,58,60,67,68] with 95% CI outside the range of the overall pooled estimate (this included the 4 studies reported in the article by Turcu-Stiolica and colleagues [31]). When these 13 outlier studies were excluded from the meta-analysis, the pooled willingness to offer PBV services slightly increased to 72.27% (95% CI: 68.09–76.09). However, between-study heterogeneity remained high at 86.1% (*p* < 0.001). Generally, the pooled willingness was 70% and over in the AMR, EMR and SEAR WHO region, respectively, and in the random sampling, influenza vaccine and low risk of bias subgroups (Table 4). The pooled willingness across the studies that reported a response rate (68.56%, 95% CI: 54.76–79.71, number of studies = 14, total pharmacists = 4753) and those that did not (70.24%, 95% CI: 61.19–77.94, number of studies = 14, total pharmacists = 4124) was not statistically significant (Q = 0.05, df = 1, *p* = 0.8282). Overall, there was no evidence (*p* > 0.05) of the moderating effect of period of publication, type of vaccine assessed, PBV service availability during study, sampling technique and the overall risk of bias classification as shown by meta-regression (*p* = 0.9871, R2 = 0%) and in Table 4.

### 3.5. Publication Bias

**Figure 4:**
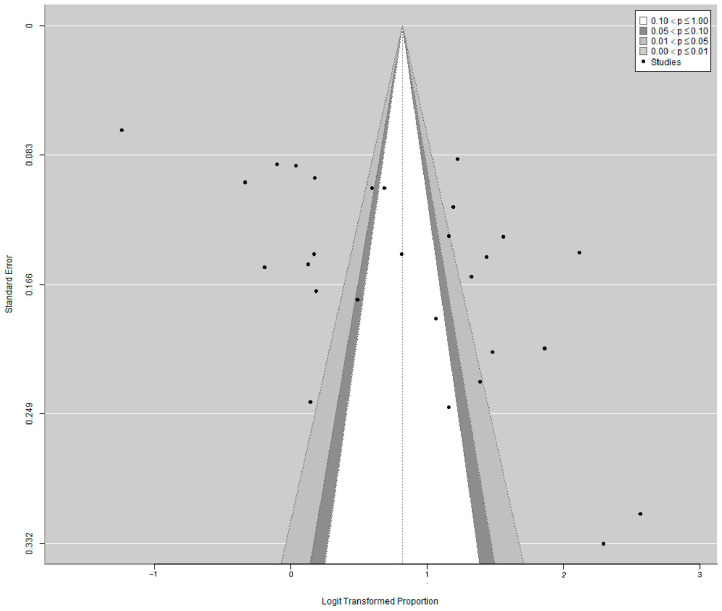
Contour-enhanced funnel plot showing asymmetry.

**Figure A2:**
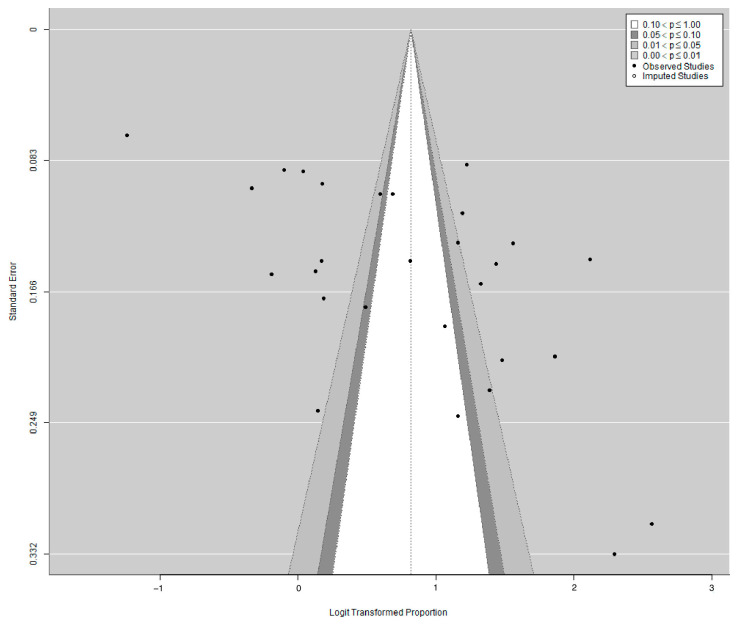
Trim-and-fill adjustment to contour-enhanced funnel plot showing no study imputed.

**Figure A3:**
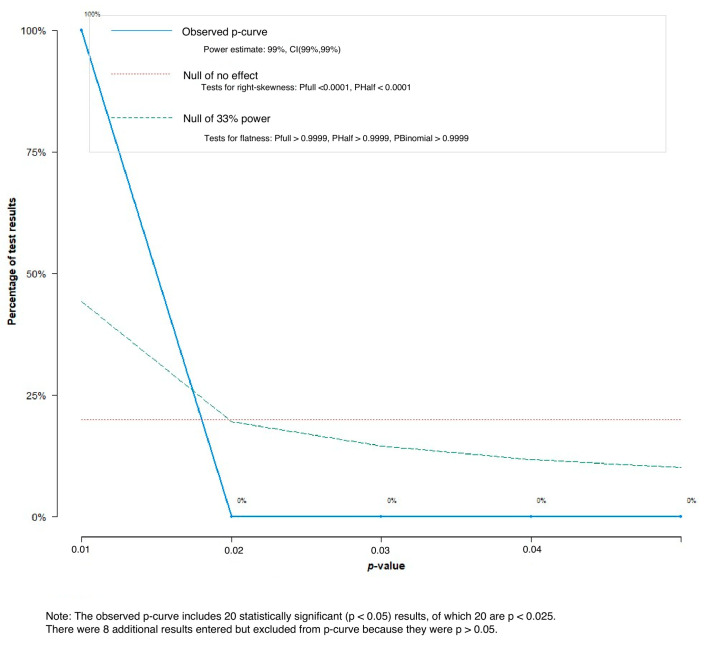
*p*-curve showing statistically significant right-skewness and non-significant test for flatness.

Visual inspection of the contour-enhanced funnel plot in Figure 4 indicated the presence of asymmetry, which was further confirmed quantitatively by the Eggers test (Intercept = 11.52, 95% CI: 5.45–17.6, *p* = 0.0009). However, trim-and-fill adjustments showed no missing study on the funnel plot, suggesting that the cause of the asymmetry is more likely due to factors other than publication bias (Figure A2). Further estimation of the pooled estimate was not performed after the trim-and-fill adjustment given that between-study heterogeneity remained high at 98%. This is because of the known limitation of the trim-and-fill method and its tendency to produce spurious results when the between-study heterogeneity is substantial [69]. Further correction via a *p*-curve indicated the presence of evidential value with a statistically significant right-skewness (pfull < 0.001) and non-significant flatness (pfull > 0.999) test that was suggestive of a true effect (Figure A3).

## 4. Discussion

To the best of our knowledge, this is the first study that has attempted to quantify the extent of pharmacists’ willingness to offer PBV services. This review’s findings show that most pharmacists are willing to offer vaccination services. However, the overall pooled vaccination willingness of approximately 70% suggests that nearly a third are not willing to offer the service. This lack of interest by a significant proportion of the workforce is an important finding that highlights the need for strategies that will enhance pharmacists’ involvement in this expanded role. Poor access to vaccines remains a public health concern and is a threat to global health security [70]. Pharmacists’ role expansion via authorization for vaccination services is a key strategy for improving vaccine access [8,9,10,11,12]. As primary healthcare providers, and the most accessible health practitioners, pharmacists are well placed to offer vaccination services [71]. Reports show that primary care pharmacists have up to 10 times more interactions with their patients compared to other providers, underscoring pharmacists’ contributions to accessible healthcare [72]. Other studies demonstrate that for some patients, pharmacists are often the first port of call for health-related concerns, especially in low- and middle-income countries [73,74]. Therefore, the lack of willingness to offer PBV services by a third of the workforce shown in the review indicates potential gaps in vaccine access, especially for populations that are increasingly reliant on community pharmacists [72,75,76].

PBV services increase vaccine uptake and coverage, especially for at-risk, hard-to-reach and vulnerable individuals [2,13,14,15,16,17,19,75]. More recently, the mobilization of pharmaceutical resources and expanded authorization for vaccination services enhanced patient convenience and increased access to lifesaving COVID-19 vaccines [8,9,10,11,12,77]. Pharmacists’ involvement in this service played a key role in containing the spread of the COVID-19 disease in high-, mid- and low-income countries alike [8,9,10,11,12,77]. Given the benefits and impact of PBV services, pharmacists’ continued involvement remains critical. It is therefore imperative to identify strategies that will ensure that pharmacists are willing, actively and continuously involved in PBV service provision. The meta-analysis finding showing broad similarities in pharmacists’ vaccination willingness across the WHO regions suggest that the disparity reported in the studies included in this review is unlikely to be solely related to peculiarities in country context. This is broadly underscored by the review finding showing willingness that also differed within the respective countries. While a few studies reported that more pharmacists in urban practices were willing to offer PBV services compared to their rural counterparts [35,59,63], the extent of this disparity in willingness could not be fully assessed via meta-analysis. This was because most of the studies in this review included a mix of pharmacists in rural and urban practice locations. Rural pharmacists are frequently involved in providing primary care and public health services [78,79]. Reports, however, suggest that workforce shortages, lack of capacity and inadequate institutional support limit involvement in these activities [80]. This feature may explain the reported lower propensity for PBV services by rural pharmacists. The lower willingness reported among rural pharmacists may also be related to the evidence that showed that pharmacists in urban areas were twice as likely to be certified for PBV services [35]. This is also underscored by the review finding showing that certification for vaccination correlates with willingness for service provision, with rural pharmacists less likely to be certified [34,36,68].

Further, this review found that PBV services were often offered below capacity [35,52], a finding that aligns with other evaluative studies reporting a relatively low annual volume of vaccination offered by the service [19,29]. This may explain the outcome of an existing review that showed only a modest to no increase in vaccination uptake with PBV service availability in the United Kingdom [17]. Reports indicate that extended opening hours correlate with the volume of immunization given within PBV services [19]. Therefore, pharmacists’ willingness to offer PBV services via extended opening hours and weekend availability as shown in this review suggests the potential for increased access to vaccines in the respective communities served [36]. This is especially important given that existing reports demonstrate that up to 50% of COVID-19 vaccines were delivered by pharmacists in the United Kingdom through a mix of appointment booking and extended opening hours [81,82]. Furthermore, pharmacists’ willingness in this review was not dependent on whether the study was conducted prior or during the COVID-19 pandemic, nor was it influenced by the specific vaccine offered within the service. However, the relatively lower proportion of pharmacists that were willing to offer the COVID-19 vaccine may allude to the reported hesitancy and misinformation about this vaccine type [83,84]. Pharmacists can play a crucial role in curbing vaccine hesitancy through the dissemination of accurate information and providing education and counseling [85]. Therefore, their continued involvement in vaccine advocacy, administration and patient counseling can positively impact uptake [18]. The lack of or limited training available for PBV services is a key barrier reported by pharmacists in this review [31,32,51,54,56,57,60]. This underscores the need for appropriate training prior to service provision. Global surveys show that only a few countries incorporate vaccination training in their undergraduate pharmacy education or post-graduate curriculum [86]. Addressing this gap in vaccination training for pharmacists is essential and could enhance willingness by increasing individual pharmacist’s confidence in their ability to provide the service [39]. Global advocacy efforts by the International pharmaceutical Federation (FIP) are already underway with the launch of the FIP Transforming Vaccinations Globally and Regionally” program [87]. National-level programs targeted towards specific pharmacy practice areas and locations showing low involvement in service provision are needed to address the observed gaps in pharmacists’ willingness.

This review had some limitations. Even though the willingness estimate suggested a trend that evidenced a true effect, the I^2^ statistic of greater than 75% indicated substantial heterogeneity. The source of this heterogeneity could not be identified or corrected via the sensitivity and subgroup analysis conducted in this review. More than half of the studies in this review employed a non-probability sampling technique and did not report the response rate. Where reported, the response rate was lower than 50% in about a third of the studies. This suggested a potential for bias, especially in relation to the precision of the estimates reported in the respective studies and the pooled estimates obtained in the meta-analysis. However, the sensitivity analysis showing there was no statistically significant difference between the studies that reported a response rate and those that did not provide confidence that the pooled estimates in the meta-analysis are robust. In addition, although pharmacists’ willingness was explicitly elicited in most of the included studies, willingness was implied in the study by Carpenter et al. and Kummer et al., which assessed pharmacists’ readiness for the service and their vaccination certification status, respectively [30,51]. These two studies may have introduced some bias, and this was controlled in the meta-analysis by reevaluating the pooled estimate with the studies by Carpenter et al. and Kummer et al. removed. This latter analysis showed there was no statistically significant difference between the estimates in the analysis with Carpenter et al. and Kummer et al. studies added or removed, further providing confidence in the pooled estimates. Nearly 40% (n = 13) of the included studies were from the Americas, with no study identified for inclusion from countries in the Western Pacific region. This means gaps remain in our knowledge on the extent of pharmacists’ willingness for PBV services in the Western Pacific region, and in countries not represented in this review.

## 5. Conclusions

The findings of this review indicate that most pharmacists are willing to offer the service. However, up to a third of the workforce are unwilling to take on this expanded role, emphasizing the need to implement strategies that will increase involvement. This may include providing education and training to enhance pharmacists’ confidence in their ability and competence to provide the service. Research studies on pharmacists’ willingness for PBV services are also needed in the countries and WHO regions not represented in this review. Further studies are needed that will provide insight and enhance our understanding of the observed disparity in willingness between pharmacists practicing in rural and urban areas, respectively. This will help inform workforce policies including advocacy and outreach strategies that will promote the further involvement of pharmacists in these distinct practice areas.

## Figures and Tables

**Table 1:** Quality assessment criteria.

Quality Criteria	Description
Sample frame	The sample frame was appropriate for the study.
Sampling process	Participants were sampled appropriately.
Sample size	Sample size was defined with justification provided.
Sample description	Sample and setting were described by the authors.
Population coverage	Population of interest appropriately covered in the identified sample.
Sample identification	Valid methods used for the identification of respondents
Outcome measurement	Pharmacist willingness was measured in a consistent way.
Statistical analysis	Appropriate statistical analysis conducted.
Response rate	Response rate was adequate (≥50%) with low response managed appropriately.

**Table 2:** Study characteristics.

Study Characteristic		Number of Studies, *n* (%)
Distribution across the WHO regions	Africa (AFR)	4 (11.8)
	The Americas (AMR)	13 (38.2)
	Southeast Asia (SEAR)	5 (14.7)
	Europe (EUR)	6 (17.7)
	Eastern Mediterranean (EMR)	6 (17.7)
Publication year	Pre-COVID-19 pandemic	15 (44.1)
	During COVID-19 pandemic	19 (55.9)
Study design	Survey	32 (94.1)
	Interviews	2 (5.9)
Sampling technique	Random	7 (20.6)
	Non-random	27 (79.4)
PBV available during study	Yes	19 (55.9)
	No	15 (44.1)
Type of vaccine assessed	Not specified	21 (61.8)
	Influenza	6 (17.6)
	HPV	1 (2.9)
	COVID-19	6 (17.6)

**Table 3:** Summary of study and findings.

Study	Country	Study Design	WHO Region	PBV Available	Sampling	Vaccine Type	Response Rate (%)	Total Sample Size (n)	n Willing (%)	Risk of Bias
Kummer 2008 [51]	USA	Survey	AMR	Yes	NRand	Non-specific	12.8	1274	286 (22)	Moderate
Marra 2010 [36]	Canada	Survey	AMR	Yes	NRand	Influenza	NR	151	123 (81.5)	Low
Pace 2010 [34]	USA	Survey	AMR	Yes	Rand	Non-specific	37.6	92	70 (76.1)	Moderate
Valiquette 2015 [37]	Canada	Survey	AMR	Yes	Rand	Non-specific	NR	115	NR	Low
Edwards 2015 [53]	Canada	Survey	AMR	Yes	NRand	Influenza	12	495	252 (52)	Low
Islam 2017 [52]	USA	Interviews	AMR	Yes	NRand	Non-specific	NR	40	NC	Low
Alsabbagh 2018 [35]	Canada	Survey	AMR	Yes	NRand	Influenza	18.4	780	603 (81.3)	Low
Balkhi 2018 [32]	Saudi Arabia	Survey	EMR	No	NRand	Non-specific	12.8	139	76 (55)	Low
Isenor 2018 [29]	Canada	Survey	AMR	Yes	NRand	Non-specific	26	168	156 (93)	Moderate
Westrick 2018 [3]	USA	Survey	AMR	Yes	Rand	Non-specific	15.5	292	236 (80.8)	Low
Agbo 2019 [56]	Nigeria	Survey	AFR	No	NRand	Non-specific	91	68	NR	Low
Kroemer 2020 [65]	France	Survey	EUR	Yes	NRand	Influenza	32	137	85 (62)	Moderate
Sepp 2020 [64]	Estonia	Survey	EUR	Yes	NRand	Influenza	NR	209	NR	Moderate
Polla 2020 [66]	Italy	Survey	EUR	No	Rand	Human Papillomavirus	70.7	389	NC	Low
Boniface 2021 [57]	Nigeria	survey	AFR	No	NRand	Non-specific	NR	148	110 (74)	Moderate
Gravlee 2021 [28]	USA	Survey	AMR	Yes	NRand	Non-specific	6.7	233	184 (79)	Low
Lindner 2021 [33]	Austria	Survey	EUR	No	NRand	Non-specific	12.3	380	314 (82.6)	Low
Merks 2021 [39]	Poland	Survey	EUR	No	NRand	Non-specific	NR	1777	NR	Moderate
Mukattash 2021 [54]	Jordan	Interviews	EMR	Yes	NRand	COVID-19	N/A	23	19 (82.6)	Low
Nurfirda 2021 [62]	Indonesia	Survey	SEAR	No	NRand	Non-specific	NR	120	NR	Moderate
Silva-Suárez 2021 [40]	Puerto Rico (USA)	Survey	AMR	Yes	NRand	Non-specific	26	302	230 (76.1)	Low
Turcu-Stiolica 2021 [31]	Serbia	Survey	EUR	No	NRand	COVID-19	32	188	102 (54.3)	Moderate
	Bulgaria	Survey	EUR	No	NRand	COVID-19	NR	168	76 (45)	Moderate
	Albania	Survey	EUR	No	NRand	COVID-19	NR	109	99 (90.8)	Moderate
	Romania	Survey	EUR	No	NRand	COVID-19	NR	171	91 (53.2)	Moderate
Youssef 2021 [59]	Lebanon	Survey	EMR	No	Rand	Adult vaccines	NR	412	274 (66.5)	Low
Youssef 2021 [38]	Lebanon	Survey	EMR	No	Rand	Influenza	NR	412	316 (76.9)	Low
Zhao 2021 [27]	USA	Survey	AMR	Yes	Rand	Non-specific	4	255	218 (75.2)	Low
Carpenter 2022 [30]	USA	Survey	AMR	Yes	NRand	COVID-19	65	69	37 (52)	Moderate
Jarab 2022 [41]	Jordan	Survey	EMR	Yes	NRand	COVID-19	N/A	201	174 (86.6)	Moderate
Ang 2022 [60]	Malaysia	Survey	SEAR	No	NRand	Non-specific	NR	492	439 (89.2)	Low
Alnahar 2022 [55]	Jordan	Survey	EMR	Yes	NRand	Non-specific	NR	403	260 (64.5)	Low
Tadele et al. 2023 [67]	Ethiopia	Survey	AFR	No	NRand	Non-specific	NR	423	230 (54.4)	Low
Chumnumwat et al. 2023 [68]	Thailand	Survey	SEAR	Yes	NRand	Non-specific	NC	405	169 (42.25)	Low
Kristina et al., 2022 [63]	Indonesia	Survey	SEAR	No	NRand	COVID-19	NR	120	96(80)	Moderate
Isah et al., 2021 [58]	Nigeria	survey	AFR	No	NRand	COVID-19	NR	509	242 (47.7)	Moderate
Qamar et al., 2022 [61]	Malaysia	Survey	SEAR	No	NRand	Non-specific	80.7	218	151 (69.3)	Low

**Key**: AMR—the Americas; EMR—Eastern Mediterranean; EUR—Europe; SEvAR—Southeast Asia; NR—not reported; NC—not clear; N/A—not applicable; NRand—non-random sample; Rand—random sample.

**Table 4:** Subgroup analysis.

Category	No. of Studies	No. of Pharmacists	% Willingness (95% CI)	I^2^ (*p*)	Between Groups Q (*p*)
All studies	28	8877	69.45 (61.58–76.33)	98.1% (<0.01)	Not applicable
All studies (outliers excluded)	15	3908	72.27 (68.09–76.09)	86.1% (<0.01)	
**WHO Region**					
Africa (AFR)	3	1080	58.71 (45.86–70.46)	94% (<0.01)	3.01 (0.5567)
The Americas (AMR)	10	3842	71.49 (53.32–84.63)	99% (<0.01)	
Southeast Asia (SEAR)	4	1235	72.91 (45.16–89.80)	99% (<0.01)	
Europe (EUR)	6	1153	67.10 (51.49–79.66)	96% (<0.01)	
Eastern Mediterranean (EMR)	5	1567	70.91 (61.41–78.87)	93% (<0.01)	
**Publication Period**					
Pre-COVID	9	3514	69.05 (49.65–83.46)	99.0% (<0.01)	0.01 (0.9526)
During COVID	19	5363	69.61 (62.23–76.11)	96.5% (<0.01)	
**PBV Availability**					
Yes	14	4988	69.83 (56.41–80.54)	98.7% (<0.01)	0.01 (0.9141)
No	14	3889	69.01 (60.33–76.53)	96.4% <0.01)	
**Sampling**					
Nonrandom	24	7669	68.41 (59.45–76.19)	98.2% (<0.01)	1.52 (0.2182)
Random	4	1208	75.17 (67.81–81.30)	85.4% (<0.01)	
**Risk of Bias**					
Low risk	15	5537	71.19 (63.64–77.73)	96.9% (<0.01)	0.25 (0.6155)
Moderate risk	13	3340	67.41 (53.11–79.07)	98.1% (<0.01)	
**Type of Vaccine**					
Non-specified	15	5367	70.56 (57.82–80.73)	98.7% (<0.01)	0.34 (0.8436)
Influenza	5	1975	70.66 (57.46–81.11)	96.7% (<0.01)	
COVID-19	8	1535	66.32 (53.60–77.05)	95.3% (<0.01)	

## Data Availability

The original contributions presented in the study are included in the article, further inquiries can be directed to the corresponding author.

## Appendix A

Search strategy on OvidSP.

Databases captured included: Journals@Ovid Full Text, Your Journals@Ovid, APA PsycArticles Full Text, CAB Abstracts, Embase, Global Health, HMIC Health Management Information Consortium, Ovid MEDLINE(R) and Epub Ahead of Print, In-Process, In-Data-Review & Other Non-Indexed Citations, Daily and Versions, APA PsycBooks, APA PsycExtra, APA PsycInfo, Social Policy and Practice.

Search
  1. vaccination.mp. [mp=ab, td, hw, ti, tx, bt, tn, ot, dm, mf, dv, kf, fx, dq, sh, de, md, sd, kw, ba, bk, ca, cl, ct, cw, yr, id, cc, ac, ip, vo, pg, jn, ja, bd, pu, ib, is, et, ar, bs, cf, dp, pj, pa, dt, rf, so, pb, pi, pl, ry, st, mo, op, os, se, ta, te, rw, nm, ox, px, rx, an, ui, ds, on, sy, tc, tm, pt] Total = 676,451
  2. willingness.mp. [mp=ab, td, hw, ti, tx, bt, tn, ot, dm, mf, dv, kf, fx, dq, sh, de, md, sd, kw, ba, bk, ca, cl, ct, cw, yr, id, cc, ac, ip, vo, pg, jn, ja, bd, pu, ib, is, et, ar, bs, cf, dp, pj, pa, dt, rf, so, pb, pi, pl, ry, st, mo, op, os, se, ta, te, rw, nm, ox, px, rx, an, ui, ds, on, sy, tc, tm, pt] Total = 200,388
  3. acceptance.mp. [mp=ab, td, hw, ti, tx, bt, tn, ot, dm, mf, dv, kf, fx, dq, sh, de, md, sd, kw, ba, bk, ca, cl, ct, cw, yr, id, cc, ac, ip, vo, pg, jn, ja, bd, pu, ib, is, et, ar, bs, cf, dp, pj, pa, dt, rf, so, pb, pi, pl, ry, st, mo, op, os, se, ta, te, rw, nm, ox, px, rx, an, ui, ds, on, sy, tc, tm, pt] Total = 510,695
  4. perception.mp. [mp=ab, td, hw, ti, tx, bt, tn, ot, dm, mf, dv, kf, fx, dq, sh, de, md, sd, kw, ba, bk, ca, cl, ct, cw, yr, id, cc, ac, ip, vo, pg, jn, ja, bd, pu, ib, is, et, ar, bs, cf, dp, pj, pa, dt, rf, so, pb, pi, pl, ry, st, mo, op, os, se, ta, te, rw, nm, ox, px, rx, an, ui, ds, on, sy, tc, tm, pt] Total = 1,713,747
  5. 2 or 3 or 4, Total = 2,329,737
  6. pharmacist*.mp. [mp=ab, td, hw, ti, tx, bt, tn, ot, dm, mf, dv, kf, fx, dq, sh, de, md, sd, kw, ba, bk, ca, cl, ct, cw, yr, id, cc, ac, ip, vo, pg, jn, ja, bd, pu, ib, is, et, ar, bs, cf, dp, pj, pa, dt, rf, so, pb, pi, pl, ry, st, mo, op, os, se, ta, te, rw, nm, ox, px, rx, an, ui, ds, on, sy, tc, tm, pt] Total = 285,519
  7. pharmacy.mp. [mp=ab, td, hw, ti, tx, bt, tn, ot, dm, mf, dv, kf, fx, dq, sh, de, md, sd, kw, ba, bk, ca, cl, ct, cw, yr, id, cc, ac, ip, vo, pg, jn, ja, bd, pu, ib, is, et, ar, bs, cf, dp, pj, pa, dt, rf, so, pb, pi, pl, ry, st, mo, op, os, se, ta, te, rw, nm, ox, px, rx, an, ui, ds, on, sy, tc, tm, pt] Total = 384,799
  8. 6 or 7, Total = 530,946
  9. 1 and 5 and 8, Total = 965
  10. remove duplicates from 9, Total = 744

Pharmacy-Related Journals Manually Searched for Relevant Articles

- Currents in Pharmacy Teaching and Learning (CPTL)
- American Journal of Pharmacy Education (ACPE)
- International Journal of Pharmacy Practice (IJPP)
- The Pharmaceutical Journal (PJOnline)
- Research in Social and Administrative Pharmacy (RASP)
- Journal of Pharmacy (JP)
- Pharmacy Education Journal (PEJ)
- Journal of Pharmacy Practice and Research (JPPR)
- Journal of Pharmacy Practice (JPP)
- MDPI Pharmacy
- Clinical Pharmacy Journal
